# Implementation practice models for development in low- and middle-income countries: systematic review of peer-reviewed literature

**DOI:** 10.1186/s12889-022-13530-0

**Published:** 2022-06-09

**Authors:** William Douglas Evans, Raquel Gerard, Lorry Symington, Hina Shaikh, Sohail Agha

**Affiliations:** 1 Milken Institute School of Public Health, The George Washington University, 950 New Hampshire Ave, Washington, DC, NW 20037 USA; 2 M&C Saatchi, United Kingdom London,; 3 Stanford Behavior Design Lab, Seattle, USA

**Keywords:** International development, global health, social and behavioral theory, health communication, social marketing, implementation science

## Abstract

**Introduction:**

This study operationally defines a relatively small, but growing field of study on implementation practice models for health behavior change in the context of international development. We define ‘implementation practice models’ as theoretical models that take a practical and practitioner-focused approach to behavior change, and we illustrate how these models have been developed and applied. The paper examines the continuum of behavioral theories and their application in the context of development programs and research in low- and middle-income countries (LMICs). We describe implementation practice models, examine how they have been used to design and evaluate theory-based interventions in LMIC, and describe the state of evidence in this field of study.

**Methods:**

The authors conducted a systematic search of the published, peer-reviewed literature following the widely accepted PRISMA methods for systematic reviews. We aimed to identify all relevant manuscripts published in the English language in health, social science, and business literature that apply implementation practice models, located in an LMIC, with a behavior change objective. We located 1,078 articles through database searching and 106 through other means. Ultimately, we identified 25 relevant articles for inclusion.

**Results:**

We found that the peer-reviewed literature on implementation practice models for development has been growing in recent years, with 80% of reviewed papers published since 2015. There was a wide range of different models revealed by this review but none demonstrated clear-cut evidence of being most effective. However, the models found in this review share common characteristics of focusing on the three central tenets of Opportunity, Ability, and Motivation (OAM).

**Conclusions:**

This review found that implementation practice models for development are a promising and growing approach to behavior change in LMICs. Intervention practice models research should be expanded and applied in new domains, such as vaccination.

**Supplementary Information:**

The online version contains supplementary material available at 10.1186/s12889-022-13530-0.

## Contributions to the literature

- We review the concept of a continuum from theory testing and development to the application of implementation practice models in development programs.
- At the theory end of the continuum, behavior change is highly complex and this is a barrier to implementation, evaluation, and building a robust evidence base in development programs.
- For program implementation in LMICs to be successful, models need to be relatively simple and easy to implement.
- We analyze the problem of developing practice-based theoretical models for implementation of development programs.
- This paper reports on a systematic review of peer-reviewed literature on implementation practice models and recommends future efforts in the field.

## Introduction

This study aims to operationally define a relatively small, but growing field of study on the development, application, and evaluation of implementation practice models for health behavior change (i.e., changes in health promoting and risk behaviors in a priority population) in the context of international development. The paper examines the continuum of behavioral theories and their application in the context of international development (i.e., efforts to develop economically disadvantaged countries and regions to empower people to improve their well-being and address the causes and effects of poverty) programs and research in low- and middle-income countries (LMICs) [1], primarily in the overall domain of public health. We review extant implementation practice models, assess their strengths and weaknesses in terms of feasibility and evaluability for a range of issues, and examine how they have been used to design theory-based interventions for development.

First, we review the concept of a continuum from theory design and testing to application of implementation practice models in development programs. At the theory design end of the continuum, behavior change is conceptualized as highly complex, and this is a barrier to implementation, evaluation, and building a robust evidence base in international development programs due to the need for relative simplicity in programs implemented in low-resource contexts [2]. Here, we argue that for implementation practice to be successful, models need to be relatively simple and easy to implement, and we identify examples of such models. This paper defines and systematically reviews the literature on such implementation practice models.

### Continuum of behavioral theory

Behavioral and social science theories or models are often multi-dimensional and complex. They typically use a set of predictors, constructs, and explanations to systematically understand what motivates behavior and, in the context of public health, how to design effective interventions using this information to change and improve health behaviors at the population level [2]. However, behavioral theories generally do not focus on resource constraints that can complicate carrying out health-promoting behaviors at the individual level. These are the types of constraints that are typically present in international development programs in LMICs, and in programs serving low-income populations in high-income countries (HICs).

Health and resource inequalities in turn make use of social and behavioral theories difficult to sustain and heavily dependent on the ability to influence knowledge and attitudes over time. Since not all groups possess the opportunity (i.e., situational conditions), ability (i.e., task knowledge) and motivation (i.e., attitudes, beliefs, norms) (OAM) to modify behaviors [3], some research offers a conceptual framework for guiding and regulating public health behaviors through tools available in education, marketing and law [4]. This framework views OAM as the key variables in behavioral choice. It posits that perceptions of self-interest and trade-offs present in the marketplace of choices constrain what interventions can do to maximize societal-level health and well-being [4].

There is a growing body of evidence that theory-based interventions are more successful in health behavior change programs compared to interventions lacking theoretical underpinnings [5, 6]. The literature on health behavior theories has given rise to wide-ranging interventions that aim to catalyze behavioral change constructs to advance health-supporting policy and programming, in areas such as health communication for Zika prevention [7], improved child health [8], and nutrition/dietetic practices [9]. Prominent frameworks include the health belief model, transtheoretical model, social cognitive theory, and social-ecological model [5], all of which involve multi-dimensional constructs such as perceived vulnerability, social norms, self-efficacy, response efficacy, decisional balancing, and context-specific circumstances that mediate behaviors and, therefore, can promote or hinder desired behavioral change [10]. The literature is expansive on the use of such theories in behavioral medicine [11], as well as targeting specific behaviors, such as tobacco use, alcohol misuse and unhealthy diets, family planning, sexual risk taking, and others that contribute to widespread morbidity and mortality [12–14].

### Theory and program implementation

The use of theories for health promotion and efforts to change unhealthy behaviors is rooted in an understanding that health and social development problems do not exist in isolation. They are a function of interacting factors – sociocultural, economic and geographic – at different levels, for example, individual, family and community (including institutional factors), that impact personal agency and individual choices and decisions [5]. Therefore, health behaviors are critically intersectional in that they cannot be understood based on one factor but rather multiple factors that merge in diverse ways in connection with micro and macro environments, race, ethnicity, gender, biology, and socioeconomic status. This is especially true with regard to access to health resources and inequalities, as exemplified by the COVID-19 pandemic [15].

The complexity of intersecting factors facing behavior change interventions makes it critically important that theory be relatively simple and easy to apply. Practitioners, especially in international development, need theories that are pragmatic and can be applied despite resource constraints and other implementation barriers that may be present in LMICs. Implementation practice models, as we describe them in this paper, attempt to demystify theory and isolate essential variables such as OAM that can be addressed in a development context.

Context is important to implementation science, in particular developing tailored program approaches and identifying and promoting evidence-based practices [16]. Current literature suggests that translating research findings to public health practice is challenging because diffusion through communication channels and social systems [17] does not always adequately consider the settings or populations in which the intervention is introduced or applied [18]. In some instances, ineffective planning and intervention and evaluation strategies, and weak or non-existent testing also make it challenging to integrate evidence-based interventions into policy and practice [19]. There is a process of diffusion from behavioral theory development and research to implementation and intervention development in varied contexts [20]. At the level of theory development and research to establish evidence that supports theory, this is important and desirable for practice-based fields such as public health.

A primary use of behavioral theories is to design health interventions that will advance positive outcomes and expand evidence-based programs through diffusion, dissemination and implementation activities [21]. However, in the context of implementation, particularly within development programs, theoretical complexity becomes a barrier to successful practice and program execution, adaptation, and evaluation [22, 23]. In this paper, we define ‘implementation practice models’ as theoretical models that take a practical and practitioner-focused approach to behavior change, provide examples of such models, and illustrate how they have been developed and applied. Implementation practice models are applied theories (i.e., they operate at the translational end of the continuum of theory development) that are relatively easy to apply in practice for program development and implementation. While there have been previous studies that address issues surrounding implementation frameworks [22], we believe this is the first study to define the concept of implementation practice models and demonstrate their use in the context of international development programs in LMICs.

Implementation practice models have a number of strengths in terms of feasibility and evaluability for a range of issues. Given the diffusion process inherent to health behavior interventions and public health, and emphasis on implementation and scaling, the literature shows that implementation practice models exist. Moreover, there is an actionable core set of principles that such models adopt, including underlying constructs of opportunity and motivation, to understand behavior and encourage behavior change.

### Implementation practice model examples

There are a number of widely known examples of implementation practice models. For example, the Fogg Behavioral Model (FBM) posits the three core elements of motivation, ability and a prompt, or trigger, “must converge at the same moment for a behavior to occur” [24]. Fogg, the creator of the FBM, also identified a range of behaviors that can be modified depending on the prompt and temporal aims of whether the behavioral change is a single event, desired over a specific period, or to be taken up indefinitely [25]. The typology organizes behaviors by goal or action gradients of whether the target behavior is new, familiar, or an existing behavior that is sought to be increased, decreased or completely stopped [25]. Although relatively new to public health applications, the FBM has been used to assess the impact of social marketing campaigns on condom use [26, 27]. The model also has prompted research on whether interventions should aim to increase motivation or ability in the uptake of health-promoting behaviors, as in the case of exploring social norms influences on modern contraception use among Nigerian women [2].

Another framework for understanding human behavior and guiding interventions is the “COM-B system.” Michie and her colleagues developed this framework in which the “COM” refers to components of capability, opportunity, and motivation (the same components, albeit worded slightly differently, included in the OAM framework) that “interact to generate behaviour that in turn influences these components” [16]. As such, one or more of the core elements can be targeted in a behavioral change intervention. The researchers also created a “behavioral change wheel” to aid in characterizing and designing interventions, assuming relevant policies and resources exist in context to enable an intervention [16]. For instance, using the wheel as a guidepost, multiple operations within the intervention, such as incentives, restrictions, and education, can be used to address the core components for a desired behavioral outcome. The COM-B mnemonic has been used to analyze barriers and facilitators for behaviors in connection with chlamydia testing [28] and postnatal lifestyle choices following diagnoses of gestational diabetes [29], as well as intervention design for hearing aid use [30]. There has been substantial use of COM-B by some international institutions, such as the World Health Organization (WHO) [31]. This study investigates the extent to which models such as COM-B have appeared in the peer-reviewed literature on behavior change in LMICs.

A third example is the EAST framework developed by the quasi-governmental Behavioural Insights Team based the in United Kingdom [32]. Taking cues from behavioral economics and psychology, “EAST” forms a mnemonic that refers to easy, attractive, social and timely as key principles to understand and encourage behavior. Finding that “policymakers and practitioners find it useful to have a simple, memorable framework to think about effective behavioural approaches” [32], the developers were inspired to simplify the longer list of Messenger, Incentives, Norms, Defaults, Salience, Priming, Affect, Commitments, and Ego (MINDSPACE) influences on behaviors [16, 32]. The UK government has suggested local officials encourage restaurants to use the EAST model to spur healthy eating behaviors [33]. It also has been used to address violence in humanitarian settings [34] and develop interventions to promote walking [35] and improve mental health [36].

One common characteristic of these models is their attention not only to individual characteristics (e.g., attitudes, beliefs, and other personal factors), but also to the intersecting environmental factors that influence behavior. In the OAM framework described earlier, implementation practice models address not only motivation (e.g., my beliefs about a behavior and intention to act), but also opportunities and ability to act in the environmental context.

The specific aim of the present study is to operationally define implementation practice models, examine how they have been applied in international development, and conduct a systematic review of the published literature in this area in accord with the Preferred Reporting Items for Systematic Reviews and Meta-Analyses (PRISMA) guidelines. To the best of our knowledge, there are no studies that have examined the literature for implementation practice models or attempted to define such models with a core set of inputs.

The main research question (RQ1) is the following: What is the extent and nature of evidence published on implementation practice models? This study further aims to investigate two hypotheses:

(H1) There are some practical implementation practice models that represent best practices and may be recommended as a basis for resources and intervention design in the context of international development.

Overall, the study will describe the state of evidence for implementation practice models in the context of international development. By describing this distinct approach to development programs, we anticipate growth of future programs that apply implementation practice models and evaluation research in this area.

## Methods

The authors conducted a systematic search of the published, peer-reviewed literature using all relevant major online research literature databases (specified below) and following widely accepted methods for systematic review [37]. We note that social and behavior change communications, social marketing, and related interventions focused on the application of implementation practice models are also widely represented in unpublished reports and other “gray” literature. However, in this study, we focus on peer-reviewed literature to ensure quality of evidence and consistency with accepted systematic review practices.

### Search Strategy

We aimed to identify all relevant manuscripts published in the English language in health, social science, and business literature that apply implementation practice models and practices, used at least one of the four Ps of marketing, and had an objective targeting promotion of behavior change. We based the review methodology in part on methodologies used in a previous review of branded social marketing campaigns conducted by the lead author [38]. Specifically, we searched the following health, social science, and business databases: PubMed, PsycINFO, Web of Science (includes Science Citation Index Expanded, Social Sciences Citation Index, and Arts and Humanities Citation Index), Communication & Mass Media Complete, Academic Search Premier, Business Source Premier, CINAHL, Health Source: Nursing/Academic Edition, and Health Source: Consumer Edition.

We selected search terms based on the authors’ experiences in the field and conducting previous reviews, and in consultation with a medical research librarian. We applied the following criteria to conduct the search: 1) limited to only include articles published from the year 2000 onward; 2) search terms included implementation, [OR] implementation model, [OR] international development [AND] behavior change, [OR] health behavior, [OR] habit, [OR] goal setting, [OR] communication, [OR] marketing, [OR] brands, [OR] branding, [OR] health promotion, [OR] disease prevention; 3) went beyond other recent reviews to include implementation practice models’ evaluation studies (to the extent of any published results) [38]; and [4] coding included population targeted, implementation methods, research/evaluation methods, outcomes (including differential effects on audiences), behavior targeted, country/region, urban/peri-urban/rural, and age range target (adolescents, young adults, older).

For completeness, we also searched literature known to the authors, including publications on implementation practice models and theories, social and behavior change communication, social marketing, and related intervention studies in LMICs and development contexts. In particular, the bibliographies of three recent meta-analyses on social marketing and mass media interventions were reviewed, and potential citations were screened following the methods described [39–41].

We searched all sources listed above in the date range of January 2000 to March 2021. The search was conducted in April 2021 using Covidence software. Based on this process, we created a Covidence database of all identified unduplicated articles on implementation practice models and programs in the peer-reviewed literature. Two reviewers reviewed all abstracts and full text articles, and their work was supervised by the lead author. Based on abstract review, we immediately excluded articles that did not relate to implementation model evaluation or programs, were clearly not original research, or did not report on any evidence for the program design (formative research) or effectiveness (evaluation).

### Screening

**Fig. 1:**
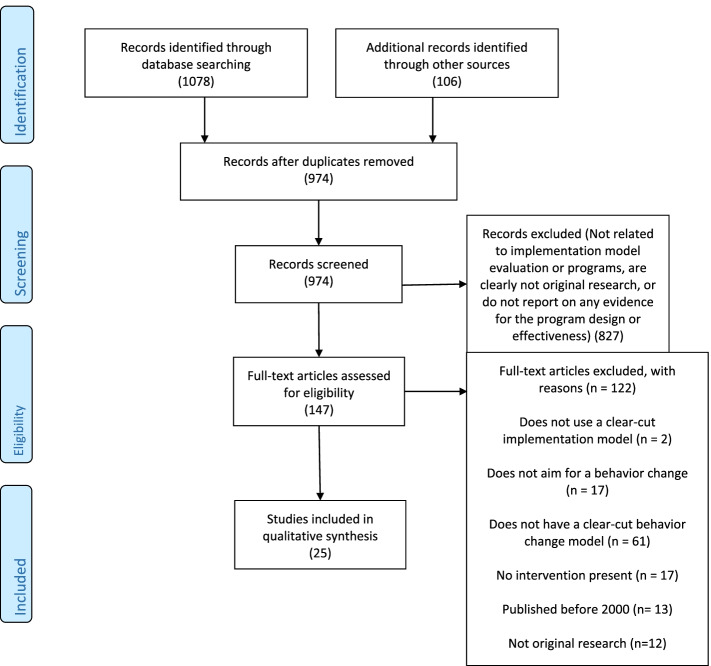
PRISMA Diagram of Systematic Review Process

Next, we obtained and reviewed all articles meeting our specific criteria for inclusion in the study. Namely, we screened them for reports on implementation practice models and programs that: (1) were original research (not review papers, meta-analyses, or commentaries); (2) utilized some form of identifiable implementation model or theory (e.g., reported on use and/or evaluation of such a model or theory); (3) targeted behavior change (not merely determinants of behavior such as knowledge, attitudes, and beliefs); and (4) targeted a specific objective based on the implementation model or theory. We also screened to ensure the articles included specific reports of evaluation or implementation of the model or theory in question, defined as coordinated efforts to promote a specific behavioral change using the model. Based on this in-depth screening process, we excluded any articles failing to meet the full article review criteria. Figure 1 summarizes the planned review process based on Preferred Reporting Items for Systematic Reviews and Meta-Analyses (PRISMA) guidelines [42]. In this review, we followed the complete 27-item PRISMA checklist [43]. We have included the checklist as an Additional file 1 to this article.

At the identification stage, we located 1,078 articles through database searching and 106 through other means (e.g., the authors’ personal experience and professional networks). After removing duplicates, we had 974 articles for abstract screening. Of these, 827 articles were excluded due to one or more of several factors including not related to implementation model evaluation or programs; clearly not original research; or did not report on any evidence for the program design or effectiveness. This left 147 articles for full text review. Of these, we excluded 122 due to one or more of several factors including did not include an implementation model (although potentially appeared to include one based on the abstract); did not report on a behavior change; or did not have an objective or outcome based on an implementation model or related theory. As a result of this screening process, 25 studies were included in the qualitative synthesis.

### Analysis

Due to the diverse nature of the literature on implementation practice models and interventions in this area and the varying methods of reporting outcomes, we did not attempt a meta-analysis of effects of reviewed interventions on behavior. Rather, the purpose of this study is to describe the nature of the implementation practice models, interventions, and literature, hopefully promoting more uniform reporting and rigorous evaluation of such efforts in the future.

Once the review sample of articles was identified, two of the authors individually read each of the articles in-depth and coded them for specific content reported in the results section. The results of all reviews were compiled and discussed by the reviewers and the lead author. Potential sources of differences in assumptions and approaches in coding articles were identified, discussed, and resolved. Reviewers ultimately reached consensus on the coding and common procedures were adopted throughout.

Additionally, risk of bias assessment was conducted for individual studies We used the revised Cochrane Risk of Bias tool (ROB 2) [44] for randomized trials, and the ROBINS-I tool [45] for non-randomized studies.

## Results

Table 1 provides a summary of basic information gleaned from each implementation model in the articles reviewed. The articles dealt with interventions relating to a wide variety of health issues and behaviors, including maternal and child health, sexual health, family planning, and nutrition. Twelve of the 25 studies (48%) focused on women as the target audience, and the remainder were divided among LGBTQ + health (3/25), men (4/25), adolescents ages 15–24 (2/25), rural (2/25), and urban populations (3/25).

**Table 1:** Characteristics of reviewed publications

Author/date	Title	Population	Region	Implementation model	Main outcomes reported	Significant Effect
Longfield 2011 [43]	Increasing safer sexual behavior among Lao kathoy through an integrated social marketing approach	LGBTQ +	Southeast Asia	PERForM	Intervention awareness/reactions; Behavioral outcomes Increased levels of condom and water-based lubricant use. Improved levels of knowledge about the importance of consistent condom use	No positive significant effect reported that could be attributed to the intervention
Meekers 2005 [44]	The impact on condom use of the "100% Jeune" social marketing program in Cameroon	Adolescent Cohort (15 to 24)	Sub-Saharan Africa	Health Belief Model, Social Learning Theory, and Theory of Reasoned Action	Pre-behavioral/intermediate outcomes; Behavioral outcomes Increased percentage of youth who used a condom during last sexual intercourse with regular partner	Significant changes in perceived condom attributes and access, self-efficacy, and perceived social support
Gutierrez 2010 [45]	Community-based prevention leads to an increase in condom use and a reduction in sexually transmitted infections (STIs) among men who have sex with men (MSM) and female sex workers (FSW): the Frontiers Prevention Project (FPP) evaluation results	LGBTQ +	South Asia	Frontiers Prevention Project	Behavioral outcomes Significant correlation between increased condom use with regular partners and lower probability of STI sero-positivity	Frontiers Prevention Project intervention demonstrated a positive significant effect for condom use with female partners and for syphilis and HSV 2 sero-positivity
Kassegne 2011 [46]	Evaluation of a social marketing intervention promoting oral rehydration salts in Burundi	Women	Sub-Saharan Africa	PERForM, PSI Behavior Change Framework	Behavioral outcomes Greater use of ORASEL and with significant improvements in perceived availability, knowledge of the signs of diarrhea and dehydration, social support, and self-efficacy	Positive significant association between ORASEL use and behavioral determinants
Wood 2012 [47]	Understanding why women adopt and sustain home water treatment: Insights from the Malawi antenatal care program	Women	Sub-Saharan Africa	The Transtheoretical Model, The Diffusion of Innovations Theory, Consumer Purchase Decision Process	Pre-behavioral/intermediate outcomes; Behavioral outcomes Increased awareness of the need to treat water, encouraged trial use, and supported continuing use	No positive significant effect reported
Agha 2021 [1]	Understanding how social norms affect modern contraceptive use	Adolescent Cohort (15 to 24)	Sub-Saharan Africa	Fogg Behavior Model	Pre-behavioral/intermediate outcomes; Behavioral outcomes Social norms that discourage contraception had a statistically significant negative association with contraceptive use	Negative statistically significant association with contraceptive use in relation to social norms that discourage contraception
Sarrassat 2015 [48]	Behavior Change After 20 Months of a Radio Campaign Addressing Key Lifesaving Family Behaviors for Child Survival: Midline Results From a Cluster Randomized Trial in Rural Burkina Faso	Women	Sub-Saharan Africa	SATURATION +	Behavioral outcomes Improvement in episodic behaviors such as care seeking for diarrhea, saving money during pregnancy, and obtaining treatment for fast/difficult breathing	Positive significant effects on care seeking behaviors
Engl 2019 [49]	CUBES: A practical toolkit to measure enablers and barriers to behavior for effective intervention design	Men	Sub-Saharan Africa	CUBES	Intervention awareness/reactions; Pre-behavioral/intermediate outcomes; Behavioral outcomes Men developed positive as well as negative beliefs, influenced by individuals around them, as they move through various stages of change	No positive significant effect reported
Ingabire 2018 [50]	Evaluation of a multi-level intervention to improve postpartum intrauterine device services in Rwanda	Women	Sub-Saharan Africa	Theory Of Planned Behavior	Intervention awareness/reactions; Behavioral outcomes Increased level of postpartum intrauterine device insertion	No positive significant effect reported
Kim 2019 [51]	A process evaluation of the quality improvement collaborative for a community-based family planning learning site in Uganda	Women	Sub-Saharan Africa	Quality Improvement Model & Collaborative Improvement Model	Intervention awareness/reactions; Behavioral outcomes Increased learning through midwife mentorships and positive trends in the number of women on a family planning service	No positive significant effect reported
Sabin 2020 [52]	Retention in hiv care among hiv-seropositive pregnant and postpartum women in uganda: Results of a randomized controlled trial	Women	Sub-Saharan Africa	WPM-based intervention, IMB model	Behavioral outcomes Retention in HIV Care for pregnant and postpartum women	No positive significant effect was reported
Coulibaly 2020 [53]	Implementing performance-based financing in peripheral health centres in Mali: what can we learn from it?	Rural	Sub-Saharan Africa	Consolidated Framework for Implementation Research	Intervention awareness/reactions; Pre-behavioral/intermediate outcomes High-performing centers exercised leadership and commitment more strongly than low-performing ones	No positive significant effect was reported
Wang 2016 [54]	The Impact of Teachers' Modifications of an Evidenced-Based HIV Prevention Intervention on Program Outcomes	Teachers	Latin America and Caribbean	Focus on Youth Caribbean	Pre-behavioral/intermediate outcomes; Behavioral outcomes Heavy modifications of FOYC lessons led to poorer student outcomes	Increased significant effect among students in the teacher groups over the 12-month follow up
Cummings 2017 [55]	A complex intervention to improve implementation of World Health Organization guidelines for diagnosis of severe illness in low-income settings: a quasi-experimental study from Uganda	Men	Sub-Saharan Africa	Behavior Change Wheel, COM-B	Pre-behavioral/intermediate outcomes; Behavioral outcomes Increased likelihood of patients being diagnosed with sepsis and severe respiratory distress	Significant increases in site-adjusted likelihood of initial assessment of temperature, heart rate, blood pressure, respiratory rate, mental status, and pulse oximetry
Johri 2020 [56]	Social and Behavior Change Communication Interventions Delivered Face-to-Face and by a Mobile Phone to Strengthen Vaccination Uptake and Improve Child Health in Rural India: Randomized Pilot Study	Rural	India	Tika Vaani model	Pre-behavioral/intermediate outcomes; Behavioral outcomes Increased levels of health knowledge	Statistical significant results were shown in higher basic health knowledge among the intervention group
Agha 2019 [25]	Use of the Fogg Behavior Model to Assess the Impact of a Social Marketing Campaign on Condom Use in Pakistan	Men	Southeast Asia	Fogg Behavior Model	Pre-behavioral/intermediate outcomes; Behavioral outcomes The odds of condom use among men with high motivation and high ability were 34 times higher than the odds of condom use among men with low motivation and low ability	Statistically significant association between self-reported condom use at least sex and categories of motivation and ability
Saggurti 2013 [57]	Effects of a health care provider intervention in reduction of sexual risk and related outcomes in economically marginal communities in Mumbai, India	Men	India	Narrative Intervention Model	Pre-behavioral/intermediate outcomes; Behavioral outcomes Patients who received treatment for gupt rog from trained providers reported receiving significantly higher rates of services than those who received services from untrained providers	Positive significant effect of the intervention in primary care settings for reducing sexual risk among married men
Sharma 2020 [58]	Evaluation of a community-based intervention for health and economic empowerment of marginalized women in India	Women	India	Community engagement model	Intervention awareness/reactions; Behavioral outcomes Increased awareness regarding maternal and child health among women	No positive significant effect was reported
Dickson-Gomez 2018 [59]	A social systems analysis of implementation of El Salvador's national HIV combination prevention: a research agenda for evaluating Global Health Initiatives	LGBTQ +	Latin America and Caribbean	Van Olmen's Health Systems Dynamic framework	Pre-behavioral/intermediate outcomes; Behavioral outcomes Improved access to HIV prevention and care	No positive significant effect was reported
Wichaidit 2019 [60]	Effect of an equipment-behavior change intervention on handwashing behavior among primary school children in Kenya: the Povu Poa school pilot study	School staff	Sub-Saharan Africa	Social Norms Theory	Behavioral outcomes Increased rates of handwashing and availability of water and soap	Probability of handwashing with soap after toileting post-intervention were significantly higher
Penn-Kekana 2018 [61]	Process evaluation of a social franchising model to improve maternal health: evidence from a multi-methods study in Uttar Pradesh, India	Women	India	Social franchising model	Intervention awareness/reactions; Behavioral outcomes Sky health providers had better knowledge and self-reported practice than comparable health providers	No positive significant effect was reported
Ma 2018 [62]	Clan-involved approaches to increasing antenatal care use in a rural minority area of China: implementation research	Women	East Asia (China, Korea, Japan, Mongolia)	Social Cognitive Theory, Diffusions of Innovations Theory, & Communication Theory	Behavioral outcomes Significant increase in awareness and uptake of antenatal care	Positive significant effect related to increased awareness of ANC
Hoddinott 2018 [63]	Nutrition behaviour change communication causes sustained effects on IYCN knowledge in two cluster-randomised trials in Bangladesh	Women	Southeast Asia	Behavior Change Communication Intervention	Behavioral outcomes Behavior change communication improved infant and young child nutrition knowledge in the first year of the intervention	Positive significant effects of levels of knowledge
Babazadeh 2019 [64]	Cognitive factors associated with brucellosis preventive behaviours among diagnosed patients: an application of Empowerment Model	Urban	MENA	Empowerment Model	Intervention awareness/reactions; Behavioral outcomes Significant effect was found on Brucellosis Preventive Behaviors by demographic variables	Level of education, knowledge, and self-efficacy were found to be positive significant predictors
Murray 2015 [65]	The Saturation + Approach to Behavior Change: Case Study of a Child Survival Radio Campaign in Burkina Faso	Women	Sub-Saharan Africa	SATURATION +	Behavioral outcomes The successful impact that the 3 principles of the Saturation + approach has on behavior change	No positive significant effect was reported. a

Only one study was published prior to 2010, and 20/25 were published in 2015 or later. The majority of studies were conducted in sub-Saharan Africa (14/25), followed by India (5/25), with others mainly conducted in the Middle East and North Africa and East Asia.

Table 1 shows the overall characteristics of the sample by variables coded in the qualitative synthesis. The main coding categories are summarized here, including the implementation model used in the study, and detailed coding results are provided in subsequent tables.

As shown in Table 2, the interventions used a wide range of intervention approaches and strategies, including mass media (radio, TV), interpersonal communication (IPC) through community outreach, and visits to households by health workers. High levels of awareness of the promoted health messages were reported. Among these, nearly all studies reviewed (24/25) reported use of some form of mass media, with the majority of these studies (17/25) using unpaid (donated) media such as radio or TV. Community outreach was the second most often reported technique (16/25), and some (9/25) studies reported community mobilization strategies (i.e., organizing members of the community to advocate for behavior change). Nearly half of the studies (12/25) used some form of mass media (paid or unpaid) and IPC. Two studies reported the use of mobile phones as a strategy. The majority of studies reported use of some kind of formative research to design and test the intervention (20/25), with in-depth interviews (IDI) being the most common (13/25). One article reported use of audience segmentation, and one used tailored messages, but in many cases, articles did not provide sufficient information to code for these specific marketing strategies (i.e., it was not reported).

**Table 2:** Intervention approaches

Author/date	Use of formative research	Intervention channels
Longfield 2011 [46]	IDI; quantitative	Unpaid mass media; posters; community outreach
Meekers 2005 [47]	Focus groups; IDI; quantitative	Paid mass media; unpaid mass media; posters
Gutierrez 2010 [48]	IDI; quantitative	Unpaid mass media; community outreach
Kassegne 2011 [49]	IDI; quantitative	Paid mass media; unpaid mass media; posters; community outreach
Wood 2012 [50]	IDI	Paid mass media; unpaid mass media; community outreach; community mobilization
Agha 2021 [27]	Quantitative	Unpaid mass media; community outreach
Sarrassat 2015 [51]	None	Paid mass media
Engl 2019 [49]	Focus groups; IDI; quantitative	Unpaid mass media; community outreach
Ingabire 2018 [50]	Focus groups; quantitative	Unpaid mass media; community outreach; community mobilization
Kim 2019 [54]	Focus groups; IDI	Unpaid mass media; posters; community outreach
Sabin 2020 [55]	None	Unpaid mass media; mobile phones
Coulibaly 2020 [56]	IDI	Unpaid mass media; community mobilization
Wang 2016 [57]	None	Community outreach
Cummings 2017 [58]	Quantitative	Unpaid mass media; community mobilization
Johri 2020 [59]	Quantitative	Unpaid mass media; community mobilization; mobile phones
Agha 2019 [26]	Quantitative	Paid mass media
Saggurti 2013 [60]	None	Community mobilization
Sharma 2020 [61]	None	Posters; community outreach; community mobilization
Dickson-Gomez 2018 [62]	IDI	Unpaid mass media; community outreach
Wichaidit 2019 [63]	IDI	Posters; community outreach
Penn-Kekana 2018 [64]	None	Paid mass media; unpaid mass media; community outreach; community mobilization
Ma 2018 [65]	Focus groups; IDI	Community outreach; community mobilization
Hoddinott 2018 [66]	Focus groups; Quantitative	Unpaid mass media; community outreach
Babazadeh 2019 [67]	IDI	Community outreach
Murray 2015 [68]	Focus groups; IDI	Unpaid mass media

Table 3 provides a summary of the study design and outcomes in the articles reviewed. Most of the articles reviewed described studies with an observational design; the remaining studies were equally split between experimental and quasi-experimental designs. Most articles reported the study sample size (22/25) and sample characteristics (e.g., demographics) (16/25). Multivariate analysis and/or path analysis was used to report statistics in 18/25 of the studies. All 25 studies aimed to assess behavioral objectives (i.e., the effort aimed to achieve such an outcome), including specific behaviors such as family planning or nutrition, and clearly stated these outcomes. A majority of articles (14/25) made clear statements about pre-behavioral objectives (i.e., the effort aimed to achieve such an outcome), including attitudes, beliefs, intentions, social norms and related predictors of behavior.

**Table 3:** Study design and outcomes

Author/date	Sampling	Research design	Statistics reported	Significant effects
Longfield 2011 [46]	Sample size; characteristics 288 surveys were administered in November 2004 and 415 surveys were administered in June 2006	Not reported	Descriptive; multivariate	Intervention awareness; behavioral
Meekers 2005 [47]	Sample size; characteristics The study was completed by 2907 15–24 years old in 200 and 3536 15–24 years old in 2002	Not reported	Descriptive; multivariate	Pre-behavioral; behavioral
Gutierrez 2010 [48]	Sample size; characteristics 12 Frontiers Prevention Project sub-sites and 12 Non-Frontiers Prevention Project sub-sites were randomly selected	Not reported	Descriptive; multivariate; path models	Behavioral
Kassegne 2011 [49]	Sample size; characteristics In 2006, 2,499/3,728 met the criteria for the study. In 2007, 2,101 / 5,408 met the criteria	Not reported	Descriptive; multivariate	Pre-behavioral; behavioral
Wood 2012 [50]	Sample size Study participants were selected from 333 women who completed a 2010 follow-up survey and their close friends and family	Not reported	Not reported	Pre-behavioral; behavioral
Agha 2021 [27]	Sample size; characteristics 1916/2051 eligible women completed interviews	Not reported	Descriptive; multivariate; path models	Pre-behavioral; behavioral
Sarrassat 2015 [51]	Sample size; characteristics 5,000 mothers of under-5 year old children	Not reported	Descriptive; multivariate	Behavioral
Engl 2019 [49]	None	Observational	Descriptive	Intervention awareness; pre-behavioral; behavioral
Ingabire 2018 [50]	Sample size; characteristics 9,020 pregnant women were counseled and 2,575 PPUIDs were inserted	Not reported	Descriptive; univariate	Behavioral
Kim 2019 [54]	Sample size 291 participants were included in the study	Not reported	Descriptive	Behavioral
Sabin 2020 [55]	Sample size; characteristics 120 pregnant women	Not reported	Descriptive	None
Coulibaly 2020 [56]	Sample size 161 semi-structured interviews, 69 informal interviews, and 96 non-participant observation sessions	Quasi-experimental	Not reported	Pre-behavioral; behavioral
Wang 2016 [57]	Sample size Data was collected in 2012 from 77 government schools from 155 teachers and 3646 students	Quasi-experimental	Multivariate	Pre-behavioral; behavioral
Cummings 2017 [58]	Sample size; characteristics 5759 eligible patients took place in the study	Quasi-experimental	Descriptive; multivariate; path models	Pre-behavioral; behavioral
Johri 2020 [59]	Sample size; characteristics 387 households (184 intervention and 203 control) were included and randomized in the study	Not reported	Descriptive; multivariate; path models	Pre-behavioral; behavioral
Agha 2019 [26]	Sample size; characteristics 617/806 men were interviewed	Quasi-experimental	Descriptive; multivariate; path models	Intervention awareness; pre-behavioral; behavioral
Saggurti 2013 [60]	Sample size; characteristics 554/736 participants completed follow up	Not reported	Descriptive; multivariate	Pre-behavioral; behavioral
Sharma 2020 [61]	Sample size; characteristics The intervention population included 37,324 participants	Not reported	Descriptive	Behavioral
Dickson-Gomez 2018 [62]	Sample size 20 members of the Country Coordinating Mechanism, 20 members of specialized clinics, 20 personnel at HIV clinics, and 28 supervisors and outreach workers were interviewed	Quasi-experimental	Not reported	Pre-behavioral; behavioral
Wichaidit 2019 [63]	Sample size; characteristics 30 schools were divided into 3 groups to obtain information for this study	Not reported	Descriptive	Behavioral
Penn-Kekana 2018 [64]	None	Quasi-experimental	Descriptive	None
Ma 2018 [65]	Sample size In-depth interviews were conducted with 40 young women, 20 husbands, 20 clan leaders, and 20 health providers	Not reported	Descriptive	Intervention awareness; pre-behavioral; behavioral
Hoddinott 2018 [66]	Sample size; characteristics 2,341 women were surveyed via 4 rounds	Quasi-experimental	Descriptive; multivariate	Pre-behavioral; behavioral
Babazadeh 2019 [67]	Sample size; characteristics 238 patients with brucellosis were recruited to answer questionnaires	Not reported	Descriptive; multivariate	Intervention awareness; behavioral
Murray 2015 [68]	None	Not reported	Descriptive	Behavioral

Also, we coded for the evidence reported by the studies reviewed. In total, 9 studies reported on intervention awareness/reactions as a measured outcome, and 5/9 report positive statistically significant effects on that outcome. Of the studies that measured pre-behavioral outcomes, such as attitudes, beliefs, and social norms, [14], all showed a positive statistically significant effect on those outcomes. Finally, 23 studies reported on behavior change as a measured outcome, and all showed a positive statistically significant effect on the targeted behavior(s). Each of these studies used self-report measures of behavior.

## Discussion

This study operationally defines a relatively small, but growing field of study on the development, application, and evaluation of practical implementation practice models for health behavior change in the context of international development. Implementation practice models, as defined, represent a practical application of behavioral theory targeted to the applied end of the spectrum of research and evidence generation in the social and behavioral sciences. These models are important because practitioners, especially in development contexts in LMICs, often face resource and other constraints and must prioritize program implementation. At the same time, design of effective programs requires use of theory. Thus, implementation practice models offer a practical approach to the use of theory in program design in development settings.

In answer to RQ1, we found that the peer-reviewed literature on implementation practice models for development, as defined, is modest, but has been growing in recent years. Most of the reviewed papers (80%) were published since 2015. A wide range of implementation practice models were reported, with no clear predominant theory or model. It is noteworthy that models identified by the authors as prominent in the implementation literature, such as the Fogg Behavior Model, COM-B, and EAST, appeared only three times in total in this review. These models have been published much more widely in high-income countries (HICs), and one recommendation from this review is that they should be considered for greater use in LMIC contexts given their published evidence of effectiveness in promoting behavior change [24, 32, 69].

This review found that the vast majority of studies using implementation practice models were effective in demonstrating self-reported behavior change, with a smaller majority demonstrating positive effects on pre-behavioral determinants (intermediate outcomes), such as attitudes, beliefs, and social norms. However, most studies did not use experimental or quasi-experimental designs, and there was a mix of more and less rigorous reporting of specific intervention strategies, sampling approaches, outcomes measures, and statistics. Overall, the literature on implementation practice models is somewhat inconsistent at this stage, and more rigorous reporting of study features and components would improve our understanding of their value.

With respect to H1 and H2, there was a wide range of different models revealed by this review, and none demonstrated clear-cut evidence of being most effective. However, the models found in this review, such as PERForM, Fogg Behavior Model, COM-B, Behavior Change Wheel, and SATURATION + , share common characteristics of focusing on the three central tenets of Opportunity, Ability, and Motivation (OAM). This shared focus on OAM represents a focus on practical application, and the simplification of behavioral theory to maximize its utility in application. The use of implementation practice models with this common approach provides a solution to the problem of complexity in behavioral theory described in this paper.

This review has some implications for the use and future development of implementation practice models as a practice-focused basis for design of evaluation studies. In particular, there is a dearth of rigorous evaluation using quasi-experimental and, where possible and desirable, experimental designs. Recognizing that resources and environmental context may not always enable such studies, more such research should be conducted to build the evidence base when circumstances permit.

While we found a high percentage of the reviewed studies reported statistically significant effects on behavior, research designs varied and relatively few were rigorously controlled. We recommend that future research focus on maximizing rigor of research designs, and to increase measurement of intervention reach, frequency, awareness, and reactions to evaluate dose–response effects of delivery. At the same time, evaluation of fidelity of the implementation based on the chosen model or theory is crucial and should become a regular feature of future studies in this area.

Additionally, future interventions and research studies should focus on implementation practice models noted at the outset of this paper, and shown effective in HICs, such as COM-B, the Fogg Behavior Model, and EAST, among others. Given that there is evidence in favor of these models, and in their own manner each apply the OAM framework, more evidence on the applicability and effectiveness of models that use opportunity, ability, and motivation as key constructs is needed. This also calls for increased focus on valid and reliable measurement of OAM variables, and development of standardized metrics in the field.

One surprising finding, given that it has been used by international institutions such as the WHO [31], was the lack of publications on interventions using COM-B in LMICs. We only found one paper (Cummings et al., 2017) [58] that reported using COM-B. This is surprising given that the model appears regularly in the peer-reviewed literature. But it appears mainly in published studies set in HICs, and also potentially in gray literature in LMICs.

This study has implications for future programming in LMICs. Specifically, implementation practice models have potential to make theory-based programs easier to develop and implement in low-resource settings, and by practitioners who do not have advanced theoretical and research training. Increasing the use of theory in interventions in LMIC has potential improve quality, increase the rigor of evaluations, and thus improve the evidence base on the effectiveness of such programs over time.

The study also has some limitations. First, terminology in connection with implementation practice models is somewhat difficult to identify in some cases due to inconsistent use of language, a phenomenon found in other fields of applied intervention such as health communication and social marketing [38, 70]. Second, we did not conduct a meta-analysis and thus cannot comment on the quality of actual data analysis or reporting of data in the reviewed papers. Finally, we acknowledge that there is substantial gray literature on implementation practice models and their application in development contexts in LMICs that are not captured in this study. For purposes of consistency and knowing the universe of articles to be screened, we elected to follow the PRISMA methodology and restrict our focus to peer-reviewed literature.

## Conclusions

This review found that implementation practice models for development are a promising and growing approach to behavior change in LMICs. The peer-reviewed literature shows that these models are generally effective in promoting behavior change, but there are relatively few rigorously controlled studies. We recommend future research focus on the role of the OAM framework and development of common valid and reliable measures. Intervention models’ research should be expanded and applied in new domains. In particular, future research should examine whether implementation practice models are effective when dealing with multidimensional behaviors requiring potentially complex decision making. One example would be vaccine hesitancy in light of the COVID-19 pandemic. Documentation of these approaches following standardized reporting will enhance growth of the field.

## Supplementary Information

**Additional file 1.** PRISMA 2020 expanded checklist.

## Data Availability

The authors declare that all datasets used and/or analysed during the current study are available from the corresponding author on reasonable request. The authors declare that they have no financial or non-financial competing interests related to this publication.

## Abbreviations

COM-B: Capability, Opportunity, Motivation-Behavior
EAST: Easy, Attractive, Social, and Timely
FBM: Fogg Behavioral Model
HICs: High Income Countries
LGBTQ +: Lesbian, Gay, Bi-sexual, Transgender, Queer
LMICs: Low and Middle Income Countries
OAM: Opportunity, Ability, Motivation
PRISMA: Preferred Reporting Items for Systematic Reviews and Meta-Analyses
